# Suppression of capsule expression in *Δlon* strains of *Escherichia coli* by two novel *rpoB* mutations in concert with HNS: possible role for DNA bending at *rcsA* promoter

**DOI:** 10.1002/mbo3.268

**Published:** 2015-09-25

**Authors:** Shanmugaraja Meenakshi, M Hussain Munavar

**Affiliations:** Department of Molecular Biology, School of Biological Sciences, Centre for Advanced Studies in Functional and Organismal Genomics, Madurai Kamaraj University [University with Potential for Excellence]Madurai, Tamil Nadu, 625 021, India

**Keywords:** Ces, HNS, *rcsA*, *rpoB*, *Δlon*

## Abstract

Analyses of mutations in genes coding for subunits of RNA polymerase always throw more light on the intricate events that regulate the expression of gene(s). Lon protease of *Escherichia coli* is implicated in the turnover of RcsA (positive regulator of genes involved in capsular polysaccharide synthesis) and SulA (cell division inhibitor induced upon DNA damage). Failure to degrade RcsA and SulA makes *lon* mutant cells to overproduce capsular polysaccharides and to become sensitive to DNA damaging agents. Earlier reports on suppressors for these characteristic *lon* phenotypes related the role of cochaperon DnaJ and tmRNA. Here, we report the isolation and characterization of two novel mutations in *rpoB* gene capable of modulating the expression of *cps* genes in *Δlon* strains of *E. coli* in concert with HNS. *clpA*, *clpB*, *clpY,* and *clpQ* mutations do not affect this capsule expression suppressor (Ces) phenotype. These mutant RNA polymerases affect *rcsA* transcription, but per se are not defective either at *rcsA* or at *cps* promoters. The results combined with bioinformatics analyses indicate that the weaker interaction between the enzyme and DNA::RNA hybrid during transcription might play a vital role in the lower level expression of *rcsA*. These results might have relevance to pathogenesis in related bacteria.

## Introduction

Studies pertaining to transcriptional regulatory mechanism that govern capsule genetics do gain medical importance as capsular polysaccharides are one of the virulence determining factors in most of the pathogenic bacteria. Capsular polysaccharides serve as an insulating coat for most of the pathogenic bacteria which in turn helps the bacteria to overcome the immune reactions that are elicited in the Human body (Clarke 2010). In the case of *Escherichia coli* the group I capsule is made of colanic acid (composed of glucose, galactose, and glucuronic acid). The genes responsible for capsule synthesis (∼19 genes) are organized as a very large cluster/operon. The genetic regulation of capsular polysaccharide synthesis in *E. coli* has been extensively studied (reviewed by Gottesman 1989; Gottesman and Maurizi 1992). Capsular polysaccharides are overproduced in the absence of Lon, which is one of the major protease found in *E. coli*. RcsA, (which is the actual substrate for Lon) along with RcsB activates *cps* genes that in turn lead to synthesis of capsule resulting in mucoid phenotype of *lon* mutant (Gottesman et al. 1985; reviewed by Majdalani and Gottesman 2006). The other prominent phenotype of *lon* mutant is the extreme sensitivity to DNA damaging agents and this stems from accumulation of another well-studied substrate of Lon viz. SulA, the cell division inhibitor which gets induced upon DNA damage. Thus, overproduction of capsule and sensitivity to DNA damaging agents are regarded as the hallmark phenotypes of *lon* mutants (reviewed by Gottesman and Maurizi 1992). Search for Alternate Lon-like proteases (Alp) led to the understanding of a cascade of events that regulate elicitation of Alp phenotype in *lon* mutants. Extensive study on the elicitation of Alp phenotype in *lon* mutants clearly reveal that in *Δlon* mutants presence of *ssrA::cat* coupled with a multicopy Kan^R^ plasmid/pUC4K or a novel mutation in *dnaJ* (*faa*) could suppress the above said characteristic phenotypes of *lon* mutants to appreciable degree; Also, when *lon* mutants bear both *faa* and *ssrA::cat* mutations, the effect was found to be even stronger (Trempy and Gottesman 1989; Kirby et al. 1994; Trempy et al. 1994; Munavar et al. 2005) (see also Discussion). Recently from this Laboratory it has been reported that mutations in *rpoB* and *gyrA* can alleviate the sensitivity selectively toward the Mitomycin C (MMC) in an SOS-deficient *E. coli* strains such as *lexA3 ind*^−^ and *recA* (Shanmughapriya and Munavar 2012). Furthermore, earlier reports also suggest that *rpoB* mutations show pleiotropic phenotype in *E. coli* (Jin and Gross 1989; Zhou and Jin 1998; Zhou et al. 2013). Taking the above points into consideration, in this investigation we sought for *rpoB* (*rif*) mutations capable of suppressing either one or both the phenotypes of *lon* mutant. In this effort, starting from a *Δlon* strain harboring *cps::lac* transcription fusion, two such *rif* alleles were isolated; among the two alleles, one of them is novel, hitherto unreported and confers slow movement to the RNA polymerase, the other one is identical to *rpoB2* reported by Yanofsky (1981) and confers fast movement. These two *rif* alleles are capable of suppressing only the overproduction of capsular polysaccharides by 50–70% compared to the parental (*Δlon*) strain (the sensitivity to DNA damaging agents of *Δlon* strain is not at all affected by these two *rif* alleles). It has been shown that this suppression by both the *rif* alleles do not stem from the global transcription defect and it arises due to selective inhibition of *rcsA*/*cpsB* transcription. The suppressor phenotype by these *rpoB* alleles are herein after referred as Ces referring to **C**apsule **e**xpression **s**uppressor. Results reported herein show that functions like ClpA, ClpB, ClpY, and ClpQ do not play a role in this Ces-mediated suppression and mutant RNA polymerase enzymes per se are not defective in initiating transcription at *rcsA*/*cps* promoters. Our results also substantiate major role for HNS in elicitation of Ces phenotype by these *rif* alleles. It is proposed that the ability of DNA sequences present upstream of *rcsA* to get bended and positions of mutations in *rpoB* mutants have impact on binding affinity of RNAP with template DNA and is of great importance.

Many of the pathogenic bacteria like *Salmonella enterica*, *Vibrio cholerae*, *Klebsiella pneumoniae*, *Erwinia amylovora*, *Proteus mirabilis,* and *Pseudomonas aeruginosa* posses homologous signaling pathway that regulate the virulence factors in these nonenteric bacteria. We have shown here that the mutation in one of the widely conserved genes (*rpoB*) among bacteria can selectively modulate/suppress capsule overproduction in concert with HNS. Our study would be highly beneficial to understand the regulation of expression of genes involved in capsule synthesis in other pathogens, considering *E. coli* as a model system.

## Experimental Procedures

### Media composition, chemicals, fine chemicals, genetic, and molecular techniques used in this study

The media (conventional Luria-Bertani and Minimal media) composition used in this entire study is essentially as described in Miller (1992). Materials used for media, buffer, solutions, most of the Antibiotics, Mac-Conkey Lactose Agar and other fine chemicals were purchased from Hi-Media, India. Streptomycin was purchased from Sarabhai Chemicals, India. Methyl methane sulfonate (MMS) was purchased from Sisco Research Laboratories Pvt. Ltd, India and the final concentration of each of them is quoted wherever appropriate. The primers used in this study were obtained from Synergy Scientific, India. All the Genetic techniques were according to Miller (1992) (with minor modifications) and molecular techniques employed in this study are as per Sambrook and Russel (2001).

### Bacterial strains, phages, plasmids, and primers used in this study

Table1 gives the list of bacterial strains, phages, plasmids, and primers used in this study. All the bacterial strains are the derivatives of *E. coli* K-12 and the Genetic nomenclature is according to Demerec et al. (1966) and Berlyn (1998).

**Table 1:** List of *Escherichia coli* strains, phages, plasmids, and primers used in this study

Strain	Relevant genotype	Source/reference/construction
SG20780	F^−^ *Δ(argF-lac)*169 *lon*510 *cpsB*10*::lac rpsL*150	S. Gottesman, NIH, USA
SG20781	F^−^ *Δ(argF-lac)*169 *lon*^+^ *cpsB*10*::lac rpsL*150	S. Gottesman, NIH, USA
CAG18618	F^−^ *λ*^−^ *rph*-1 *thiC3178::Tn10kan*	Lab Collection
HR318	F^−^ *λ*^−^ *rph*-1 *btuB::Tn10 rpoB8*	R. Harinarayanan, CDFD, India
HR318K	Same as HR318 but has *thiC3178::Tn10kan*	This Study HR318 X P1/(CAG18618)
MMR6	Same as SG20780 but has *thiC3178::Tn10kan rpoB12*	This Study
MMR23	Same as SG20780 but has *thiC3178::Tn10kan rpoB77*	This Study
MMR8	Same as SG20780 but has *thiC3178::Tn10kan rpoB8*	This Study SG20780 X P1/(HR318K)
KL226	HfrC*(*PO2A*) relA1 spoT1*	Laboratory Collection
SMM12	Same as KL226 but has *thiC3178::Tn10kan rpoB12*	This Study KL226 X P1/(MMR6)
SMM23	Same as KL226 but has *thiC3178::Tn10kan rpoB77*	This Study KL226 X P1/(MMR23)
SMM8	Same as KL226 but has *thiC3178::Tn10kan rpoB8*	This Study KL226 X P1/(HR318K)
AB1157	F^−^ *thr-1*, *araC14*, *leuB6*(Am), *Δ(gpt-proA)62*, *lacY1*, *glnX44*(AS)*, hisG4*(Oc), *rpoS396*(Am), *rpsL31*(Str^R^), *argE3*(Oc), *thi-1*	Laboratory Collection
DM49	Same as AB1157 but has *lexA3 Ind*^−^	Laboratory Collection
DM49RN	Same as DM49 but has *zfa*723::*Tn10 gyrA87 argE*^*+*^ *rpoB87*	Shanmughapriya and Munavar (2012)
SM49AK	Same as DM49RN but has *clpA::kan*	Shanmugapriya (2013)
SM49BK	Same as DM49RN but has *clpB::kan*	Shanmugapriya (2013)
SM49QC	Same as DM49RN but has *clpQ::cat*	Shanmugapriya (2013)
SM49YC	Same as DM49RN but has *clpY::cat*	Shanmugapriya (2013)
MMR6A	Same as MMR6 but has *clpA::kan*	This Study MMR6 X P1/(SM49AK)
MMR6B	Same as MMR6 but has *clpB::kan*	This Study MMR6 X P1/(SM49BK)
MMR6Q	Same as MMR6 but has *clpQ::cat*	This Study MMR6 X P1/(SM49QC)
MMR6Y	Same as MMR6 but has *clpY::cat*	This Study MMR6 X P1/(SM49YC)
MMR23A	Same as MMR23 but has *clpA::kan*	This Study MMR23 X P1/(SM49AK)
MMR23B	Same as MMR23 but has *clpB::kan*	This Study MMR23 X P1/(SM49BK)
MMR23Q	Same as MMR23 but has *clpQ::cat*	This Study MMR23 X P1/(SM49QC)
MMR23Y	Same as MMR23 but has *clpY::cat*	This Study MMR23 X P1/(SM49YC)
SMM780A	Same as SG20780 but has *clpA::kan*	This Study SG20780 X P1/(SM49AK)
SMM780B	Same as SG20780 but has *clpB::kan*	This Study SG20780 X P1/(SM49BK)
SMM780Q	Same as SG20780 but has *clpQ::cat*	This Study SG20780 X P1/(SM49QC)
SMM780Y	Same as SG20780 but has *clpY::cat*	This Study SG20780 X P1/(SM49YC)
SMM781A	Same as SG20781 but has *clpA::kan*	This Study SG20781 X P1/(SM49AK)
W3110	F^−^*λ*^−^*IN(rrnD-rrnE)1 rph-1*	Laboratory Collection
ZK819	Same as W3110 but has *Δ(argF-lac)*169 *rpoS819 bgl*^–^ *hns::kan*	S. Mahadevan, IISc,India
SMM780H	Same as SG20780 but has *hns::kan*	This Study SG20780 X P1(ZK819)
SMM781H	Same as SG20781 but has *hns::kan*	This Study SG20781 X P1(ZK819)
MMR6H	Same as MMR6 but has *hns::kan*	This Study MMR6 X P1(ZK819)
MMR23H	Same as MMR23 but has *hns::kan*	This Study MMR23 X P1(ZK819)
SMM780R	Same as SG20780 but bearing pHYD535	This Study
SMM781R	Same as SG20781 but bearing pHYD535	This Study
MMR6R	Same as MMR6 but bearing pHYD535	This Study
MMR23R	Same as MMR23 but bearing pHYD535	This Study
SMM780RC	Same as SG20780 but bearing pCL1920	This Study
SMM781RC	Same as SG20781 but bearing pCL1920	This Study
MMR6RC	Same as MMR6 but bearing pCL1920	This Study
MMR23RC	Same as MMR23 but bearing pCL1920	This Study
SMM780T	Same as SG20780 but bearing p*λ*TR1	This Study
SMM781T	Same as SG20781 but bearing p*λ*TR1	This Study
MMR6T	Same as MMR6 but bearing p*λ*TR1	This Study
MMR23T	Same as MMR23 but bearing p*λ*TR1	This Study
SMM780TC	Same as SG20780 but bearing pKK232-8	This Study
SMM781TC	Same as SG20781 but bearing pKK232-8	This Study
MMR6TC	Same as MMR6 but bearing pKK232-8	This Study
MMR23TC	Same as MMR23 but bearing pKK232-8	This Study

Phage	Relevant genotype	Source/reference
P1	*vir*	Laboratory Collection; originally obtained from N Willets, U.K.
Plasmid		
pHYD535	Derivative of pCL1920 bearing wild-type *rpoBC*^*+*^ alleles cloned under *lac* promoter	R. Harinarayanan, CDFD, India
pCL1920	pSC101 *ori*, Spec^R^	R. Harinarayanan,CDFD, India
p*λ*TR1	Derivative of pKK232-8 bearing *λ*TR1 site cloned under *tac* promoter	A. R. Rahmouni, CNRS, France
pKK232-8	pBR322 *ori*, Amp^R^, Cam^R^	A. R. Rahmouni, CNRS, France

Primer	Sequence	Source/reference
*rif* For	5′-CGTCGTATCCGTTCCGTTGG-3′	Shanmughapriya and Munavar (2012)
*rif* Rev	5′-GGCAACAGCACGTTCCATACC-3′	Shanmughapriya and Munavar (2012)
*cpsB* RT For	5′-GATCTCACCATGCTGCAA-3′	This Study
*cpsB* RT Rev	5′- GTCTTCATCGGCAATCAC-3′	This Study
*rcsA* RT For	5′- ACCGTTGA TGACCTTGC-3′	This Study
*rcsA* RT Rev	5′- GCCAAGGATATCGTCGAG-3′	This Study
*dnaE* RT For	5′-GATGATCGATGGCCTG-3′	This Study
*dnaE* RT Rev	5′-GAGATCAGCAACGTCAG-3′	This Study
*rpsG* RT For	5′-GTCGCGTCAT TGGTCAG-3′	This Study
*rpsG* RT Rev	5′- CGAGAGCTACTTCGAATGCT-3′	This Study
*rcsA* CL For	5′-CGGGATCC C TGA TGC TGG CGT TA-3′	This Study
*rcsA* CL Rev	5′-ACGCGTCGAC GGCATCAGGACGGTATC-3′	This Study

### Isolation and genetic characterization of *rpoB* mutations

Different aliquots of the overnight culture of SG20780 were plated on LB plates containing rifampicin (50 *μ*g/mL) and the plates were incubated at 30°C for 2–3 days. As the intention was to isolate only Cps::Lac^−^ colonies, the cells were plated on LB plates with Rifampicin and the Rif^R^ mutants obtained on LB were checked for their phenotype on Mac-Conkey Lactose Agar plates and those colonies that exhibited white/more or less white (Lac^−^) phenotype were picked and were reconfirmed. Eleven such mutants were isolated out of 367 Rif^R^ colonies obtained from 10 independent experiments and they were named as MMR1,2,5,6, MMR21–26, and MMR210. To mobilize the *rpoB* allele into various strain backgrounds, the strain CAG18618 carrying *thiC3178::Tn10kan* marker located near *rpoB* region was used. In order to confirm that the suppression is only due to mutation in *rpoB*, the *rpoB* region from all the putative Cps::Lac^−^ Rif^R^ mutants were mobilized back to the parental strain SG20780 with a nearby selectable marker *thiC3178::Tn10kan* through P1 transduction.

### Physiological characterization and complementation studies of *rif* mutants

Growth properties at different temperatures of the selected *rif* mutants were analyzed by checking their relative viability (by sequential spotting analyses of the serially diluted relevant cultures) and incubating at appropriate temperatures. The dominant and recessive natures of the *rif* mutations were determined by introducing a plasmid clone pHYD535 (pCL1920 carrying wild-type *rpoBC*^*+*^ operon cloned at *Hind*III restriction site under *lac* promoter). The transformation was carried out according to Sambrook and Russel (2001). The transformants were selected on LB Agar plates containing Spectinomycin (50 *μ*g/mL) and checked for the Rif^R^/Rif^S^ phenotype by sequentially spotting the appropriate dilutions of the relevant cultures on LB plates with Spectinomycin (50 *μ*g/mL) and IPTG (1 mmol/L) and also on LB plates with Spectinomycin (50 *μ*g/mL), Rifampicin (50 *μ*g/mL) and IPTG (1 mmol/L). The Cps::Lac phenotype was also checked by plating them on Minimal M9 plates with Spectinomycin (50 *μ*g/mL), IPTG (1 mmol/L) and X-gal (20 *μ*g/mL) (see text).

### Sequence analyses

Whole genomic DNA from all the 11 rifampicin-resistant mutants was isolated by following Chen and Kuo (1993). Then it was treated with RNase at 37°C for 30 min followed by heat inactivation at 65°C for 15 min. This genomic DNA was then used as template for the amplification of *rif* region spanning about 777 bp covering all the *rif* clusters of *rpoB* gene. The sequences of the primers are given in Table1. The conditions for the PCR amplification are as follows: 5 min at 94°C, 30 sec at 95°C, 40 sec at 50°C, 30 sec at 72°C, 2 min at 72°C for 30 cycles and maintained at 4°C. The PCR products were purified with Fermentas gel purification kit following the protocol given by the manufacturer and the samples were sequenced by Chromous, Biotech., India. With the help of NCBI-BLAST Analyses Tool, sequence analyses was carried out by comparing our sequenced data with that of *E. coli* K12 substrain MG1655 and screened for the presence of any base change/mutation manually.

### Determining the termination efficiency of *rpoB* mutations

The vector pKK232-8 carries Amp^R^ as selection marker and *cat* gene (Cam^R^) cloned under IPTG-inducible *tac* promoter. The derivative of pKK232-8, p *λ*TR1 bears *λ*TR1 region which is at *Sal*I restriction site in between the *tac* promoter and *cat* gene. The control plasmid does not contain this *λ*TR1 region. Both the plasmids were transformed into the mutants as well as to the parental strains to compare the termination efficiencies of mutant and wild-type RNAP enzymes. This was carried out by sequentially spotting different dilutions of the selected transformants on LB plates with Ampicillin (100 *μ*g/mL) as well as LB plates with Ampicillin (100 *μ*g/mL) and Chloramphenicol (100 *μ*g/mL) with IPTG (1 mmol/L). The *rif* allele(s) that renders the relevant transformants, resistant to Chloramphenicol is regarded as the one(s) with faster elongation rate and capable of transcribing past terminator.

### Beta-galactosidase assay

Overnight cultures of 0.1 mL of each strain (carrying *cps::lac* fusion) were subcultured into 5 mL of M9 minimal medium containing glucose as carbon source and grown at 30°C. The cultures were allowed to attain mid-log phase, then the OD (Optical Density) of the cultures were recorded at 600 nm wavelength. When *β*-gal was analyzed from *lac* operon, the relevant cultures were grown in minimal M9 medium containing glycerol as the sole carbon source and then the cells were induced with IPTG (Isopropyl-beta D-1-thiogalactopyranoside) (1 mmol/L) for 30 min prior to assay. *β*-galactosidase assay was carried out as described in Miller (1992).

### RT-PCR and densitometry analyses

Total RNA was isolated from each of the cultures grown till mid-log phase using RNA isolation kit from Ambion, Life Technologies, USA. The obtained total RNA was then subjected for DNase treatment (Ambion, Life Technologies, USA). The concentration of the total RNA was normalized by checking their OD at 260 nm. With the help of gene-specific primers, respective mRNAs were converted into cDNA followed by amplification using one-step RT-PCR kit (Invitrogen, Life Technologies, USA). The primer sequences used for the amplification of respective genes are given in Table1. The conditions for the cDNA preparation and PCR amplification are as follows: 30 min at 55°C, 5 min at 94°C, 30 sec at 95°C, 40 sec at 52°C, 30 sec at 72°C, 2 min at 72°C for 25 cycles and maintained at 4°C. Absence of DNA was once again confirmed by performing the same reaction without RT enzyme but with Taq polymerase. The densitometry scanning was performed with the software Image J, freely available at http://rsbweb.nih.gov/ij/download.html.

### *rcsA* cloning

Genomic DNA was isolated manually by following Chen and Kuo (1993) from a wild-type strain MG1655. It was then treated with RNase at 37°C for 30 min followed by heat inactivated at 65°C for 15 min. This preparation was used as template for the PCR amplification of *rcsA* gene. The sequences of the primers are given in Table1. The conditions for the PCR amplification are as follows: 5 min at 94°C, 30 sec at 95°C, 40 sec at 56°C, 30 sec at 72°C, 2 min at 72°C for 30 cycles and maintained at 4°C. Amplification of ∼1.2 kb fragment was confirmed by running it on 0.8% agarose gel and the clone was constructed by ligating the *rcsA*^*+*^ allele with the pBR322 using *BamH*I and *Sal*I restriction enzymes. The clone was confirmed by appropriate restriction digestion Analysis.

### Bioinformatics analyses

The software Bend.it available at http://hydra.icgeb.trieste.it/dna/index.php was used to predict the bending and curving nature of relevant genes. Approximately, 1000 base pairs upstream to the promoter of each gene were given as input and the resulting output was analyzed accordingly. The red and green colors of peak show the curving and bending natures, respectively. The structural predictions were made by using PYMOL software with the help of already available structures at PDB (PDB ID 4IGC and 2OGJ). The mutant forms were modeled using the mutagenesis option available in PYMOL.

## Results

### Isolation and characterization of *rpoB* (*rif*) mutations capable of suppressing *cps::lac* expression in *Δlon* strains

The *E. coli* strain SG20780 constructed by Gottesman and Co-workers harbors the *Δlon-*510 mutation and *cps::lac* transcription fusion (Trisler and Gottesman 1984; Brill et al. 1988). This strain appears red in color on Mac-Conkey Lactose Agar plates or blue on LB/Minimal Agar plates with X-gal due to the overexpression of *cps::lac* fusion by stabilized RcsA. As SulA is also stabilized due to absence of Lon, the strain SG20780 becomes sensitive to UV, MMS etc., (reviewed by Gottesman and Maurizi 1992). In an attempt to isolate suppressors for these phenotypes, a collection of (367) spontaneous rifampicin-resistant mutants of SG20780 from 10 independent experiments were isolated. Among the 367 *rif* mutants, 11 *rif* mutants exhibited more or less white phenotype (Lac^−^) on Mac-Conkey Lactose Agar plates and one of them was resistant to MMS (see Table S1). However, through genetic analyses it is confirmed that this MMS^R^ phenotype was not due to the *rpoB* mutation and therefore was not investigated further. In order to confirm that, it is the relevant *rpoB* mutation present in each of the mutants is responsible for the Lac^−^ phenotype, wild-type *rpoB*^*+*^ allele from CAG18618 strain (that harbors *thiC3178::Tn10kan)* was mobilized into all the 11 *rif* mutants by P1 transduction and our results clearly indicate that it is indeed the *rpoB* mutation in each of the case that elicited this phenotype; this is because, in every cross all the Rif^R^ transductants exhibited the expected Lac^−^ phenotype, whereas all the Rif^S^ transductants reverted to Lac^+^ phenotype, as like parent SG20780 (Table S2).

### The 11 Rif^R^ mutants define only two alleles; both are dominant and one hitherto unreported

**Figure 1:**
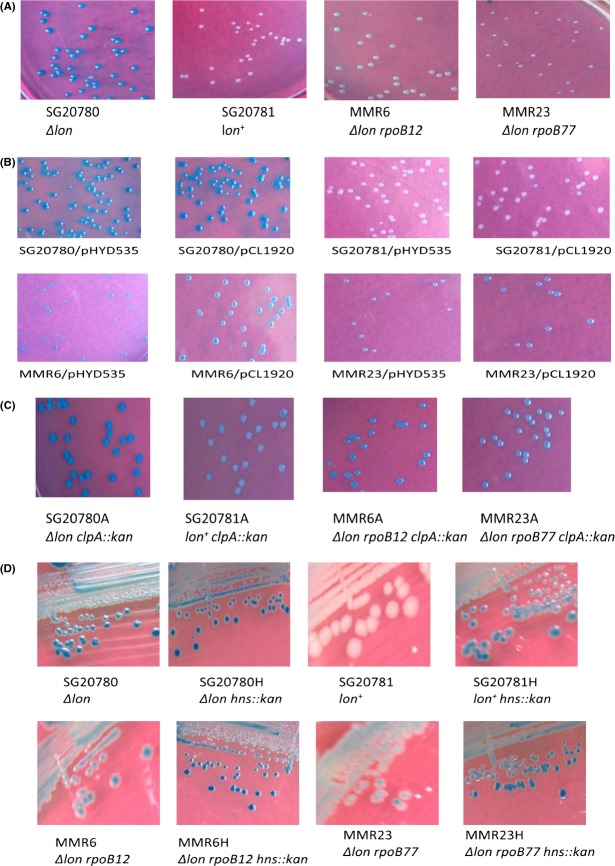
Pictures of the petri plates showing the Cps::Lac^+/−^ phenotype due to different levels of expression of *cps::lac* fusion in relevant strains. (A) Growth of *lon*^*−*^ *cps::lac* (SG20780) *lon*^*+*^ *cps::lac* (SG20781), MMR6 (*lon*^*−*^ *cps::lac rpoB12*), and MMR23 (*lon*^*−*^ *cps::lac rpoB77*) strains. Appropriate dilutions of cells were plated on relevant minimal agar plates containing X-gal (20 *μ*g/mL) and pictures were taken after ∼36 h of incubation at 30°C. (B) Growth of strains as indicated in (A) bearing the *rpoBC*^+^ clone (pHYD535) and the control plasmid vector (pCL1920). Cells were pated on similar minimal agar plates but Spectinomycin (50 *μ*g/mL) and IPTG (1 mmol/L) were added as the plasmid confers Spec^R^ phenotype and the *rpoBC*^+^ is cloned under *lac* promoter. Pictures were taken as in (A). The presence of *rpoBC*^+^ clone did not change the *cps::lac* phenotype of any strain indicating the dominant nature of both *rif* alleles. (C) Growth indicating the Cps::Lac phenotype of the strains as indicated in (A) (SG20780, SG20781, MMR6, and MMR23) bearing *clpA::kan* insertion. The cells were plated on relevant minimal agar plates containing Kanamycin (50 *μ*g/mL) and X-gal (20 *μ*g/mL).The pictures were taken after ∼44 h incubation at 30°C as the growth rate of *clpA::kan* derivatives was less compared to that of the original strains. (D) Growth indicating the Cps::Lac phenotype of the strains (SG20780, SG20781, MMR6, and MMR23) as indicated in (A) bearing *hns::kan* insertion. The cells were streaked on LB plates containing Kanamycin (50 *μ*g/mL) and X-gal (40 *μ*g/mL) as the *hns::kan* derivatives were relatively very slow grower in minimal agar plates. The pictures were taken after ∼30 h incubation at 30°C.

Earlier reports convey that most of the rifampicin-resistant mutations were found to be present in the three clusters (I, II, and III) of *rpoB* gene (Jin and Gross 1988, 1989). Therefore, the 777 bp region of *rpoB* that covers all the three clusters of *rpoB* gene was amplified. Sequence analyses of all the 11 *rif* mutations revealed the presence of only two *rif* alleles; Among the two, one defines C_**1576**_ to T (CAC to TAC) transition changing amino acid His_**526**_ to Tyr and this is essentially same as that of *rpoB2*, isolated and reported by Yanofsky and Horn (1981). The other one harbors C_**1535**_ to A (TCT to TAT) transversion changing the amino acid Ser_**512**_ to Tyr; this *rpoB* allele could perhaps be a novel one as such an allele has not been reported by others earlier. These *rif* alleles are referred to as *rpoB12* and *rpoB77,* respectively. The mutant with *rpoB12* allele, as reported earlier, is temperature sensitive at 42°C, but the mutant bearing *rpoB77* allele grows well at 42°C. The *rpoB12* allele bearing strain becomes sensitive to LB devoid of salt at all temperatures, whereas *rpoB77* mutant grows well in LB devoid of salt at 30°C and 37°C, but it becomes sensitive at 42°C (Fig. S1) (the media/salt-dependent temperature-sensitive phenotype of these strains and the defect in macromolecular synthesis in those conditions are being investigated in detail in another line of study). Both the *rpoB* alleles are dominant over the wild-type *rpoB*^*+*^ allele, as the introduction of the plasmid clone bearing the *rpoBC*^*+*^ alleles namely pHYD535 (Sarkar et al. 2013) (derived from pCL1920) into *rpoB* mutants, did not change the Cps::Lac^−^ phenotype of the strains. Figure1A and B clearly show the Lac^+^/Lac^−^ phenotype of all relevant strains.

### Evidence that *rpoB12* defines a fast moving RNAP, whereas *rpoB77* defines slow moving one

**Figure 2:**
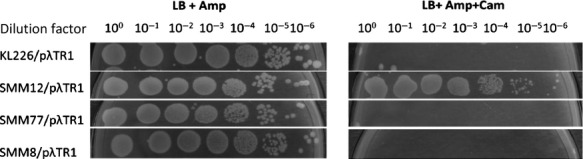
Determination of Termination efficiency of *rpoB* mutants by sequential spotting analyses of relevant strains. The overnight cultures of the relevant strains were serially diluted and relevant dilutions were spotted on LB Agar plates with Ampicillin (100 *μ*g/mL) as well as on LB plates with Ampicillin (100 *μ*g/mL), Chloramphenicol (100 *μ*g/mL), and IPTG (1 mmol/L). The plates were incubated at least for ∼24 h at 30°C. The ability of relevant strains with appropriate clone to grow both in Ampicillin and Chloramphenicol plates is the indication of fast movement of the RNAP of the relevant strain (transcription past terminator). KL226 is the WT strain, whereas SMM12, SMM77, and SMM8 are the *rpoB12*, *rpoB77,* and *rpoB8* derivatives of KL226. The fast movement for *ces12* is evident from the picture.

*λ*TR1 serves as one of the best site to study the termination efficiency in a Rho-dependent manner. Termination efficiency of different *rpoB* alleles has been determined mostly through in vitro techniques. One of the in vivo methods to determine termination efficiency is the assay of IPTG induced *β*-galactosidase enzyme from *lac* operon in a time-dependent manner which indirectly means the time taken for the formation of functional *β*-galactosidase enzyme (Jin et al. 1988, 1992). Here, we have used a vector p*λ*TR1 derived from pKK232-8 carrying a selection marker Amp^R^ and a gene coding for Chloramphenicol acetyl transferase enzyme which is cloned under IPTG-inducible *tac* promoter; In this construct, the region between the *tac* promoter and *cat* gene is interrupted by *λ*TR1 site (Guérin et al. 1998). In order to determine the termination efficiency of the *rpoB* mutations, we compared the viability of the mutants carrying the vector p*λ*TR1 on LB plates containing only Ampicillin (100 *μ*g/mL) and also LB plates with Ampicillin (100 *μ*g/mL) and Chloramphenicol (100 *μ*g/mL) along with IPTG (1 mmol/L). The *rpoB12* mutant grew very well compared to wild type confirming the earlier reports that it is able to read through the terminator site and therefore conferring fast movement to the RNAP (Jin et al. 1988; Jin et al. 1989); On the other hand, the *rpoB77* mutant did not grow on similar plates showing the inability to read through the terminator site that indirectly means that it confers slow movement to RNAP (see Fig.2). These results clearly reveal that it is not the fast/slow movement of RNAP that is responsible for the elicitation of this Ces phenotype.

### Quantification of suppression of *cps::lac* expression in *rpoB* mutants

**Figure 3:**
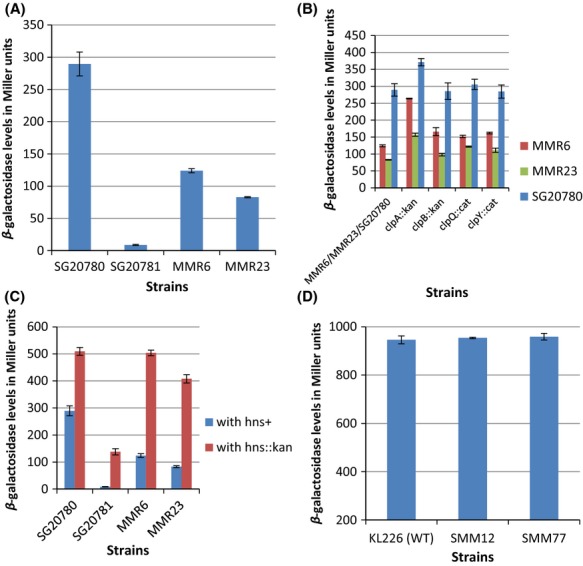
Quantification of *cps::lac/lac* operon expression through *β*-galactosidase assay in relevant strains. (A) Level of *cps::lac* expression in *rpoB* mutants along with the *lon*^−^ and *lon*^+^ controls, SG20780 and SG20781, respectively. (B) Effect of *clpA*,*clpB*, *clpQ,* and *clpY* mutations on the *cps::lac* expression in *Δlon rpoB* mutants. (C) Effect of *hns::kan* mutation in modulation of capsule expression in *rpoB* mutants. (D) Level of IPTG induced *β*-galactosidase expression from *lac* operon in relevant strains. In each case the values given are the average of three independent experiments with standard error mean. Whenever the *β*-galactosidase assay for *cps::lac* expression was perofrmed, the *lon*^−^ and l*on*^+^ strains were taken as controls. Therefore, the values given for the parental strains (*lon*^−^ and *lon*^+^) are the average of more than three experiments (for more details see text).

**Figure 4:**
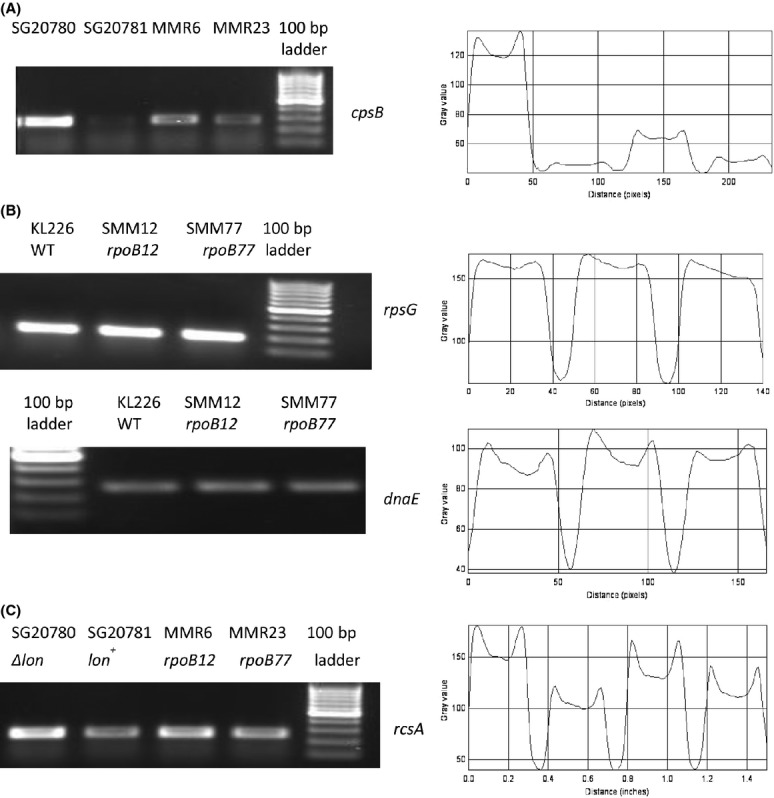
RT-PCR-based transcription profile of indicated genes and densitometry analysis of the gel pictures. (A) Transcription profile of *cpsB* gene in *rpoB* mutants with SG20780 (*lon*^−^) and SG20781 (*lon*^+^) as controls. (B) Transcription profile of house-keeping genes namely *rpsG* and *dnaE* in KL226 (WT), SMM12 (*rpoB12*), and SMM77 (*rpoB77*) strains. (C) Transcription profile of *rcsA* in *rpoB* mutants with the respective controls. In each case, 1 *μ*g of total RNA was used for RT-PCR and gel picture given for each case is the representative of three independent experiments. Shown in the side is the densitometry analysis of the respective gel pictures.

The *cps::lac* transcriptional fusion not only helps one to infer expression of *cps* genes qualitatively on relevant plates based on Lac phenotype but also makes even the quantification of *cps* expression easier by *β*-galactosidase assay. To quantify the level of expression of *cps::lac* fusion in *rpoB* mutants, *β*-galactosidase in relevant strains were assayed. The Figure3A clearly shows that *rpoB12* suppresses the expression of *cps::lac* by ∼50% and in *rpoB77* the suppression is even better (∼70%) compared to the parental strain SG20780 (*lon cps::lac rpoB*^*+*^). One of the semiquantitative methods, reverse transcription-PCR was employed to further confirm our observation. As was expected, the RT-PCR analysis of *cpsB* gene expression was in agreement with our view that *rpoB* alleles are indeed able to reduce the transcription level of *cpsB* and it is very much clear from the Figure4A.

### Selective modulation of expression of *cps::lac* by *rpoB* alleles

Many reports indicate that mutations in *rpoB* gene show pleiotropic phenotype in *E. coli* (Jin and Gross 1989; Zhou and Jin 1998; Zhou et al. 2013). As the mutations are located in the gene that code for one of the subunit of essential transcriptional machinery, it could be possible that the reduction in the level of *cps* expression might stem from the defect in global transcription by these mutant RNA polymerases with either RpoB12 or RpoB77 *β* subunit. In order to check this possibility, it is mandatory to check the expression levels of other candidate genes in *rpoB* mutants. In this respect, it was intended to analyze the expression levels of candidate genes such as *lacZ* from *lac* operon, *rpsG* coding for a ribosomal protein, and *dnaE* coding the DNA polymerase III *α* subunit. Therefore, each of the *rif* alleles (*rpoB12*/*rpoB77*) was introduced into a wild-type strain KL226 with a nearby selectable kanamycin-resistant marker (*thiC3178::Tn10kan)* and the expression level of *lac* operon was measured through *β*-galactosidase assay after IPTG induction. The results presented in the Figure3D clearly show that the level of expression of *lac* operon remains almost equal in all the cases. The expression levels of other candidate genes were analyzed through semiquantitative RT-PCR method. The expression levels of *rpsG* and *dnaE* do not vary between wild-type and the *rif*-mutant strains (see Fig.4B). From these results, it is tenable to conclude that these *rif* alleles do not interfere with the global transcription but selectively modulate *cps* expression.

### Effect of mutations in *clpA*, *clpB*, *clpY*, and *clpQ* in elicitation/modulation of Ces phenotype

In *E. coli,* it has been reported earlier that the substrates of Lon are also recognized by other protease like ClpYQ under certain conditions. Munavar et al. (2005) showed that the Lon substrate SulA is indeed degraded by ClpYQ protease in phenotypically Alp strains. Wu et al. (1999) have also shown that when ClpYQ is present in multicopy, could also degrade RcsA (Wu et al. 1999; Munavar et al. 2005). Therefore, it was necessary to check the possibility of interference of the proteases/components of the same in this suppression phenomenon. Hence, the effect of *clpA*, *clpB*, *clpQ*, *clpY* mutations on elicitation of Ces phenotype was investigated. To enable the same the insertional mutations of the above said genes were introduced into relevant strains and the level of expression of *cps::lac* was measured in each strain through *β*-galactosidase assay. The results presented in the Figure3B provide strong evidence that the studied functions do not play a role in this *rpoB*-mediated suppression. However, introduction of *clpA::kan* allele did increase the level of *β*-galactosidase to some level, but this increase was seen even in the parental strains (see also Fig.1C for the Cps::Lac phenotype). These results give a clue that ClpA might be involved in degradation of either RcsA or Cps::Lac fusion protein, a notion to be vindicated (Wickner et al. 1994; Gottesman et al. 1998).

### Transcription profile of *rcsA* in these *rpoB* mutants

To further study the molecular details of this suppression, the expression level of *rcsA* was analyzed*,* as RcsA is the positive regulator of *cps* genes (Stout et al. 1991; Ebel and Trempy 1999). It has already been reported that RcsA forms complex with RcsB that in turn regulates *cps* expression (Stout et al. 1991; reviewed by Majdalani and Gottesman 2006). The expression level of *rcsA* was analyzed through semiquantitative RT-PCR method and the gel picture (see Fig.4C) of the same which clearly shows that the level of transcription of *rcsA* indeed goes down in these *rpoB* mutants compared to the parental strain SG20780. Therefore, the reduction in transcription of *rcsA* should be playing a vital role in reducing the level of *cps* expression in these mutants.

### Effect of multicopy *RcsA* on Ces phenotype

**Figure 5:**
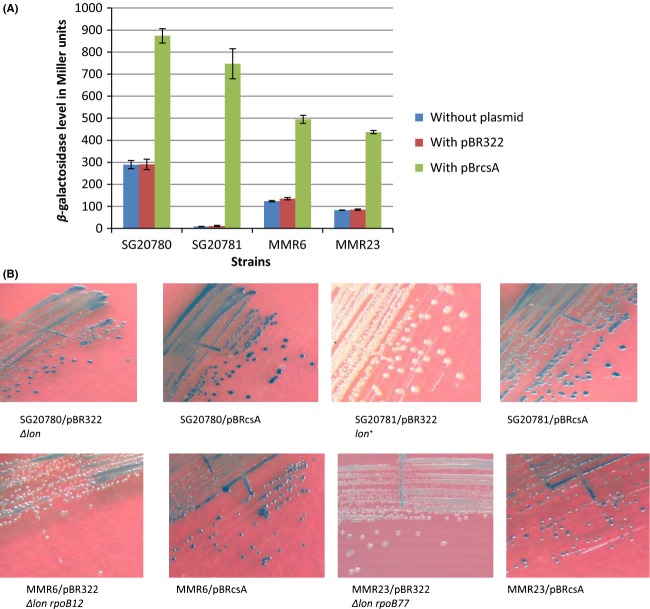
The *cps::lac* expression in relevant strains after the introduction of multicopy *rcsA*^+^ clone. (A) Effect of multicopy *rcsA*^*+*^ allele in the modulation of *cps::lac* expression in *rpoB* mutants along with the parental strains. The values given are the average of three independent experiments with standard error mean. (B) Growth indicating the Cps::Lac phenotype of the relevant strains (SG20780, SG20781, MMR6, and MMR23) bearing *rcsA*^*+*^ clone. The cells were streaked on LB agar plates containing Streptomycin (100 *μ*g/mL), Ampicillin (100 *μ*g/mL), and X-gal (40 *μ*g/mL). The pictures were taken after ∼36 h incubation at 30°C.

It has been well studied and established that overproduction of RcsA leads to high-level expression of *cps::lac* in *lon* mutants (Torres-Cabassa and Gottesman 1987; Kuo et al. 2004). The expression profile of *rcsA* in the *rpoB* mutants revealed that there is a decrease in the transcript level of *rcsA* in *rpoB* mutants compared to the parental strains. If the reduction in *rcsA* transcription alone were to be the reason for the elicitation of Ces phenotype, then the introduction of multicopy *rcsA*^*+*^ clone should theoretically abolish the phenotype of Ces in these mutants. The wild-type *rcsA* allele was cloned in pBR322 under its native promoter and was introduced into the *rpoB* mutants as well as to the parental strains. The *cps::lac* levels in these strains were determined through *β*-galactosidase assay. The Figure5A clearly shows that the beta-galactosidase levels from *cps::lac* indeed increased as expected both in parental strains and in the mutants; but this increase in level in the case of mutants are not equal to the parental *Δlon* strain. These results hint the possibility that beyond the role of RcsA there could be some other factors/mechanism might play a role in elicitation of Ces phenotype by *rif* alleles. Figure5B shows the Cps-Lac phenotype of the relevant strains.

### Mutant RNA polymerase per se is not defective at *rcsA*/*cps* promoters

The one more possibility for the reduced expression levels of *cps*/*rcsA* genes could be due to the inability of the mutant RNA polymerases with either RpoB12 or RpoB77 *β*-subunits to carry out successive transcription at *cps*/*rcsA* promoters. It has been already shown that HNS functions as repressor for *rcsA* and a small RNA *dsrA* is also involved in the regulation of *rcsA* transcription by binding to HNS there by relieving the repression by HNS (Sledjeski and Gottesman 1995). These are the known modes by which expression of *rcsA* is getting regulated in *E. coli*. If the mutant RNAPs with each of the *rpoB* mutations per se is not defective in transcription at *cps*/*rcsA* promoter, then knocking off the repressor HNS should increase the transcription level of *rcsA*. On the other hand, if these mutant RNAPs are not efficient in transcribing at *cps*/*rcsA* promoter, then even after the absence of functional HNS it should decrease the level of *rcsA* transcription. The HNS null mutant, *hns::kan* (Harwani et al. 2012) was introduced into the *rpoB* mutants as well as to the parental strains. As expected, the *cps::lac* levels were increased in the parental strains with *hns::kan*, but surprisingly, the *cps::lac* levels were higher in *rpoB* mutants with *hns::kan* which could be seen from the results presented in the Figure3C. In fact, this is very evident from the phenotype of relevant strains itself (see Fig.1D). From these results, it is tenable to conclude that these *rif* mutations do not make the RNAP defective at *cps*/*rcsA* promoters. Both these mutant enzymes indeed can transcribe the genes *cps*/*rcsA* efficiently but probably the interaction of HNS with the mutant RNAPs (with RpoB12/RpoB77 *β* subunit) leads to the elicitation of Ces phenotype.

### Possible role of bending/curving ability of DNA sequence in the promoter region affected by *rpoB* alleles

**Figure 6:**
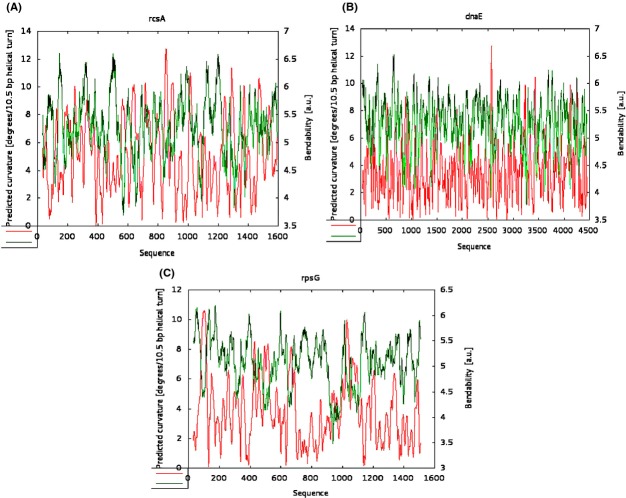
The bioinformatics analyses to predict the bending and curving abilities of relevant genes. The freely available software Bend.it was used to analyze the bending or curving ability of the upstream sequences of the relevant genes. (A) *rcsA* (B) *dnaE* and (C) *rpsG*. The red color indicates the curving ability, whereas the green color indicates the bending ability (refer text for more details).

HNS is one of the nucleoid-associated proteins and work with HNS is gaining momentum in recent years (Atlung and Ingmer 1997; Browning et al. 2010; Rimsky and Travers 2011). Extensive studies on HNS have revealed that it binds to DNA sequence preferentially to curved regions and there are also reports indicating that even binding of HNS might bend the DNA region if that particular sequence is amenable for bending (Atlung and Ingmer 1997; Shin et al. 2005; Kahramanoglou et al. 2011). As this study indicates that *hns* mutation abolishes Ces phenotype, it is conceivable that interaction of HNS with the mutant RNAP should be playing a role in elicitation of Ces phenotype. These results made us to analyze the nature of DNA sequence present in the upstream of *rcsA* gene, which is shown to be under the control of HNS. The sequence nature for some of the control genes whose expression is not altered by these *rpoB* alleles was also analyzed. Approximately 1000 base pairs upstream to the promoter of each candidate genes were taken to analyze the bending/curving ability. As could be seen from the Figure6A, it is in agreement with our view that in the case of *rcsA*, the ability for the DNA sequence to get bended/curved is higher compared to the control genes (Fig.6B and C). In the case of *dnaE*, even though our results predict that there is a probability to get bended/curved, it is well known that this gene is not under the control of HNS.

## Discussion

As could be seen from the introduction, *E*. *coli lon* mutants overexpress capsular polysaccharides due to stabilization of RcsA and become sensitive to UV/MMS due to stabilization of SulA (reviewed by Gottesman 1989, 1996; Gottesman and Maurizi 1992; Majdalani and Gottesman 2006; Clarke 2010). These two characteristic *lon* mutant phenotypes are suppressed to appreciable degree in phenotypically Alp strains (∆*lon ssrA*::*cat*/pUC4K, *lon faa* and *lon faa ssrA*::*cat*). Although in *lon ssrA*::*cat* mutants, the multicopy Kan^R^ plasmid pUC4K leads to full suppression of *lon* phenotypes, per se this Alp Helper Activity (AHA) was found to be innocuous in ∆*lon* strains bearing *ssrA*^+^ allele. That is in this original Alp strain, inactivation of *ssrA* is mandatory for elicitation of Alp phenotype and AHA only adds to this. However, *faa* mutation per se can elicit Alp phenotype in ∆*lon* strains and in such *lon faa* mutants *ssrA* has additive effect. *faa* mutation was perhaps the first identified novel allele of *dnaJ* implicated in *lon* suppression (G to A transition affecting codon 232 changing amino acid Gly to Asp). It is now well known that in these phenotypically Alp strains heat-shock induction and expression of ClpYQ protease is implicated in the degradation of SulA and models have been proposed for RcsA inactivation/degradation. In addition, in *lon faa* mutants, synthesis of SulA also has been shown to be decreased to some degree (Trempy and Gottesman 1989; Kirby et al. 1994; Trempy et al. 1994; Munavar et al. 2005). Mutations in *β* subunit of RNA polymerase affect a variety of phenotypes in *E. coli* (Jin and Gross 1989; Zhou and Jin 1998; Zhou et al. 2013). We have also reported that combination of *rpoB87* and *gyrA87* mutations permit the expression of *uvrB* by circumventing the super repression posed by *lexA3* and this expressed UvrB makes the *lexA3 rpoB87 gyrA87* strain to become resistant to MMC (Shanmughapriya and Munavar 2012). In this investigation, *rpoB* mutations that could suppress either one or both the *lon* phenotypes were sought for. Two such mutations were isolated (*rpoB12* and *rpoB77*) but both of them could suppress only one of the two hallmark phenotypes of *lon,* that is, overproduction of capsular polysaccharides. While *rpoB77* is a novel hitherto unreported allele *rpoB12 is* identical to *rpoB2* reported earlier by Yanofsky and Horn (1981). Genetic analyses confirmed that both the *rpoB* mutations could elicit the capsule expression suppressor phenotype (Ces), but to varying degrees. The physiological characterization revealed that *rpoB12* makes the cells temperature sensitive, whereas *rpoB77* allows the cells to grow well at 42°C. The interesting observation was that the *rpoB12* allele render the cells sensitive to LB devoid of salt at all temperatures. However, cells bearing *rpoB77* allele become sensitive to LB devoid of salt only at 42°C. Introduction of wild-type *rpoB*^*+*^ allele into these mutants revealed that both *rpoB* alleles are dominant over the wild-type allele. The quantification of extent of suppression showed that *rpoB12* can suppress the *cps::lac* expression to ∼50%, while *rpoB77* suppresses to ∼70%. It should be noted that ClpYQ has been shown to degrade SulA in phenotypically Alp^+^ strains and both SulA and RcsA when present in multicopy (Wu et al. 1999; Munavar et al. 2005). Therefore, the involvement of components of proteases like ClpA, ClpB, ClpY, and ClpQ in elicitation of Ces phenotype was studied. The genetic analyses reported herein revealed that these functions do not play any role in the elicitation of Ces phenotype. The peculiar observation was that the introduction of *clpA::kan* insertion increased the *β*-galactosidase levels from *cps::lac* fusion to some extent even in the parental strains SG20780 and SG20781, which gives a clue that ClpA might play a role either in the degradation of RcsA or Cps::Lac fusion protein. However, this view should be treated conjectural till proven by biochemical means.

As *β* subunit being the very crucial subunit of the essential transcription machinery RNAP, one can imagine that the low-level expression of *cps::lac* might result from global transcription defect also. However, the expression levels of other candidate genes clearly indicate that perhaps it is not due to global transcription defect but could perhaps be specific to *cps::lac*. Expression levels of *rcsA* in these *rpoB* mutants indicate that the extent of transcription of *rcsA* indeed goes down in both mutants. Taken together, it is tenable to propose that these mutant RNAPs might be defective at either *rcsA* or *cps* promoters. In order to verify this view, the repressor of *rcsA* viz. HNS was knocked-off and the expression levels of *cps::lac* in these *rpoB hns::kan* strains were checked. However, the results reported herein indirectly imply that the RNAPs are not defective per se at *rcsA* promoter. Moreover, even after the introduction of *rcsA*^*+*^ allele in multiple copies, the *cps::lac* expression levels in both *rpoB12* and *rpoB77* mutants were not equivalent to that of the parental *lon* mutant, suggesting the involvement of hitherto unidentified players/mechanism in the elicitation of Ces phenotype besides *rcsA*. Studies on HNS and other nucleoid-associated proteins are gaining momentum these days (Atlung and Ingmer 1997; Browning et al. 2010; Rimsky and Travers 2011). It has been reported that HNS preferably binds to a curved region or sometimes binding of HNS itself bends the region, which is considered to be the mode of regulation of certain genes by HNS (Atlung and Ingmer 1997; Shin et al. 2005; Kahramanoglou et al. 2011).

**Figure 7:**
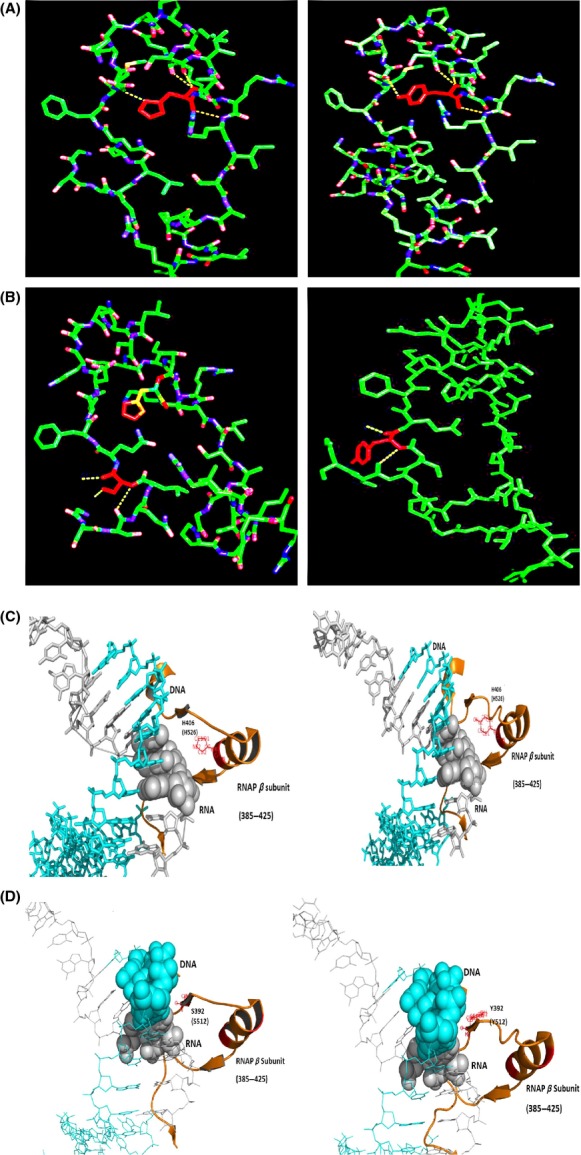
The structural analyses of mutant RpoB region of the *rpoB* mutants. Comparison of RpoB region (residues 507–563) from the mutant MMR6 (His526 to Tyr526) with that of wild type. (A) Comparison of RpoB region (residues 507–563) from the mutant MMR23 (Ser512 to Tyr512) with that of wild type. (B) Interaction of RpoB region from the MMR6 mutant (residues 385–425 in *Thermus thermophilus*) with the template DNA–RNA hybrid. (C) Interaction of RpoB region from the MMR23 mutant (residues 385–425 in *T. thermophilus*) with the template DNA–RNA hybrid. The structural prediction was made with PYMOL software. By taking the available structures in PDB (ID 2O5J and 4IGC) as template, we modeled and predicted the possible differences in the interaction with the DNA–RNA hybrid compared to the wild type. In the case of (A and B), the relevant residues are shown in red color and the yellow dotted line indicates the polar interactions with nearby residues. In the case of (C and D), the orange color represents the *β*-subunit (residues 385–425 of *T. thermophilus*, the position in *Escherichia coli* for the relevant residue is given in brackets). Cyan represents DNA, whereas gray represents RNA (refer text for more details).

In the case of *rcsA*, the exact binding sequence for HNS has not been reported so far. From this study, it could be suggested that the regulation of *rcsA* by HNS should also involve the same bending mechanism. Landick et al. (1990) have reported earlier that when a fast moving RNAP encounters competitors during initiation, it undergoes successive abortive transcription. It could be hypothesized that when a fast moving RNAP with *rpoB12* mutation encounters a DNA bending molecules such as HNS, it might undergo abortive transcription which could be the possible explanation for the lower level expression of *rcsA*. But, results reported herein clearly indicate that both fast and slow moving RNAPs could elicit Ces phenotype. Therefore, bioinformatics analyses were carried out to check the structural change(s) in binding affinity to DNA template in these mutant RpoB subunits with the help of PYMOL software by taking the already available and reported structures as templates (PDB ID 4IGC and 2O5J) (Vassylyev et al. 2007; Murakami 2013). The Figure7A and B compares the structure of the region of wild type and mutant *E. coli* RpoB subunits corresponding to amino acid residues 507–536. It can be observed that change in amino acid at position 526 from Histidine to Tyrosine in the case of *rpoB12* mutant results in change in the polar interaction with the nearby residues compared to the wild-type structure. Similarly, at the position 512, the amino acid Serine got mutated to Tyrosine in the *rpoB77* mutant and in this case also there is change in the polar interaction between the nearby residues compared to the wild type. The Figure7C and D show the interaction of the mutant RpoB subunit region of *Thermus thermophilus* (amino acid range 385–425) with the template DNA–RNA hybrid. It can be clearly seen that, when His526 is present, the atom exposed toward the template DNA–RNA hybrid is Nitrogen that exhibit positive charge which probably has higher affinity toward the DNA–RNA hybrid, but when His526 is changed to Tyr526, the exposed atom is OH which is already negatively charged that might have lesser affinity toward the DNA–RNA hybrid. In the case of *rpoB77,* the negatively charged cloud is higher compared to the wild type which might result in the lesser affinity of the RNAP with the template DNA. Therefore, we believe that these mutant RNAPs might have lesser affinity to template DNA–RNA hybrid during transcription compared to wild type and thus we propose that in this situation when these mutant RNAPs encounters bending agents like HNS, probably it tends to undergo successive abortive transcription by slipping off from the template.

To the best of our knowledge, this is perhaps a maiden report in which *rif* (*rpoB*) mutations are shown directly to affect expression of *cps* genes. In this study, although it has been shown that transcription level of *rcsA* is getting reduced in the case of *rpoB12 and rpoB77* mutants, the levels of reduction in *rcsA* alone could not account for the 50–70% reduction in level of *cpsB* expression. Therefore, it is believed that in addition to the reduction in the level of *rcsA* there may be some other unidentified factors/mechanisms which might lead to the low-level synthesis of capsular polysaccharides in these *rpoB* mutants. Detailed study currently underway in the laboratory might probably help us to understand the actual mechanism by which these *rpoB* mutations elicit the suppression of capsule expression reported herein.

## Supporting Information

**Figure S1.** Physiological characterization of the relevant strains at appropriate conditions. (A) Sequential spotting analyses of the relevant strains showing the growth pattern at 30°C and 42°C on LB agar plates containing appropriate antibiotics. (B) Sequential spotting analyses of the relevant strains showing the growth pattern at 30°C and 42°C on LB agar plates devoid of salt. (C) Sequential spotting analyses of the relevant strains showing the dominant/recessive phenotype on LB agar plates with and without Rifampicin. These experiments were performed more than twice and the pictures given are the representative for each of them (refer text for details).

**Table S1.** Distribution of Cps::Lac^−^/MMS^R^ phenotype among spontaneous Rif^R^ mutants isolated from SG20780 (*Δlon cps::lac*).

**Table S2.** Percentage distribution of Cps::Lac^+/−^ – Rif^R/S^ phenotype among Kan^R^ transductants obtained in the P1 transductional crosses involving P1 made of CAG18618 bearing Kan^R^ (*thiC3178::Tn10kan)* as donor and Rif^R^ mutants of SG20780 as recipients.
