# Supplementary figures and images for: Suppression of capsule expression in Δlon strains of Escherichia coli by two novel rpoB mutations in concert with HNS: possible role for DNA bending at rcsA promoter
Source: Microbiologyopen. 2015 Sep 25;4(5):712–29. doi: 10.1002/mbo3.268 (PMC4618605; doi:10.1002/mbo3.268)

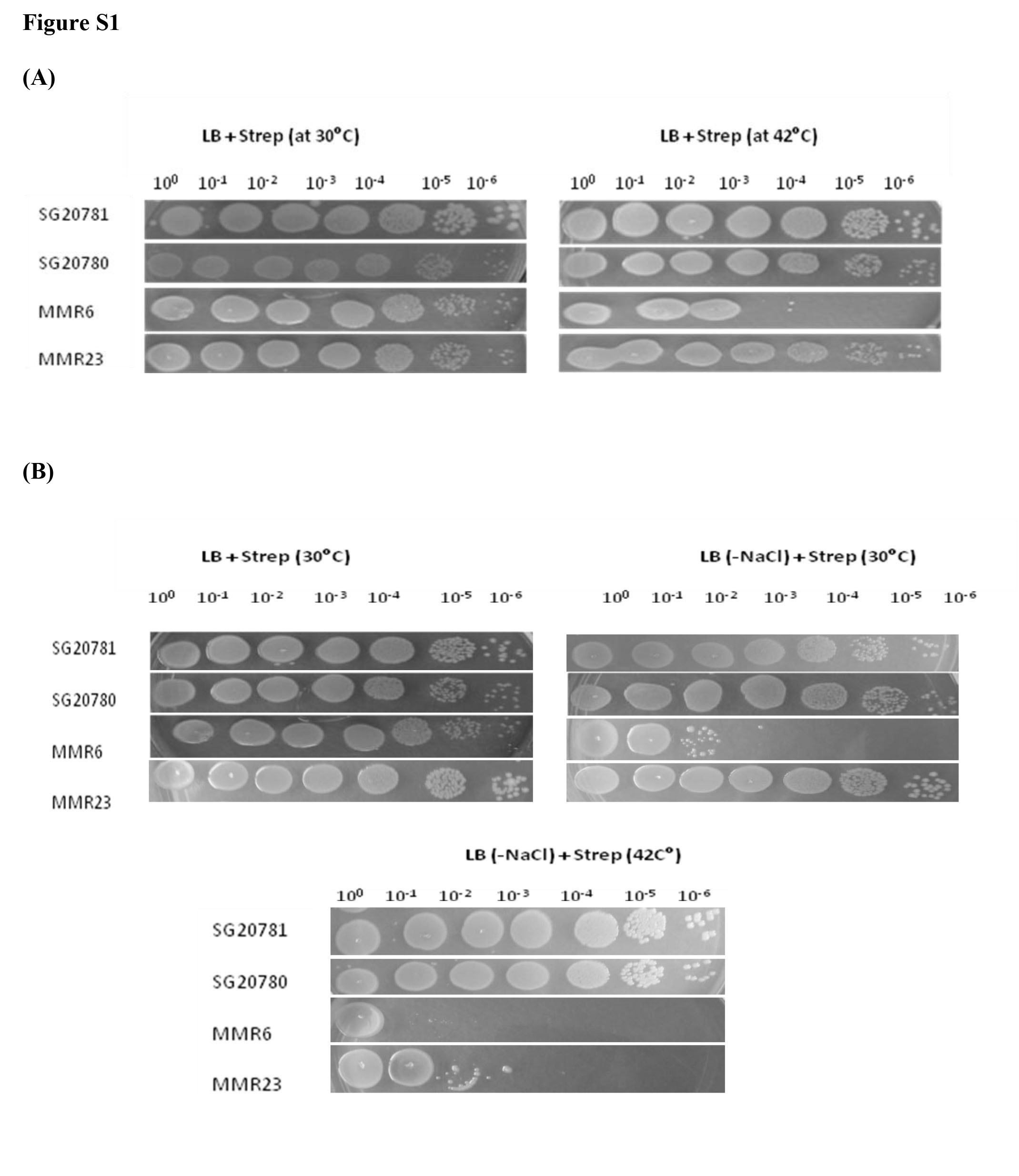

Supplement: Supplementary file 1 — Figure S1. Physiological characterization of the relevant strains at appropriate conditions. (A) Sequential spotting analyses of the relevant strains showing the growth pattern at 30°C and 42°C on LB agar plates containing appropriate antibiotics. (B) Sequential spotting analyses of the relevant strains showing the growth pattern at 30°C and 42°C on LB agar plates devoid of salt. (C) Sequential spotting analyses of the relevant strains showing the dominant/recessive phenotype on LB agar plates with and without Rifampicin. These experiments were performed more than twice and the pictures given are the representative for each of them (refer text for details). [file mbo30004-0712-sd1.jpg]

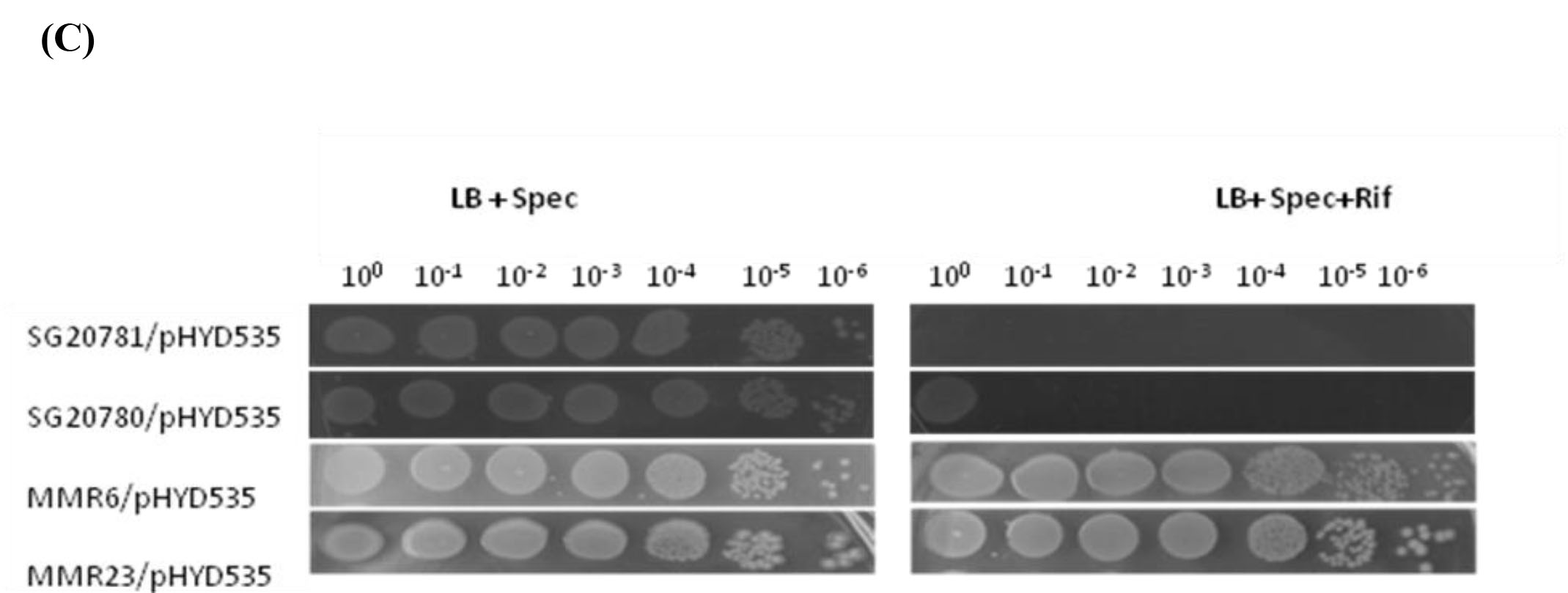

Supplement: Supplementary file 2 [file mbo30004-0712-sd2.jpg]

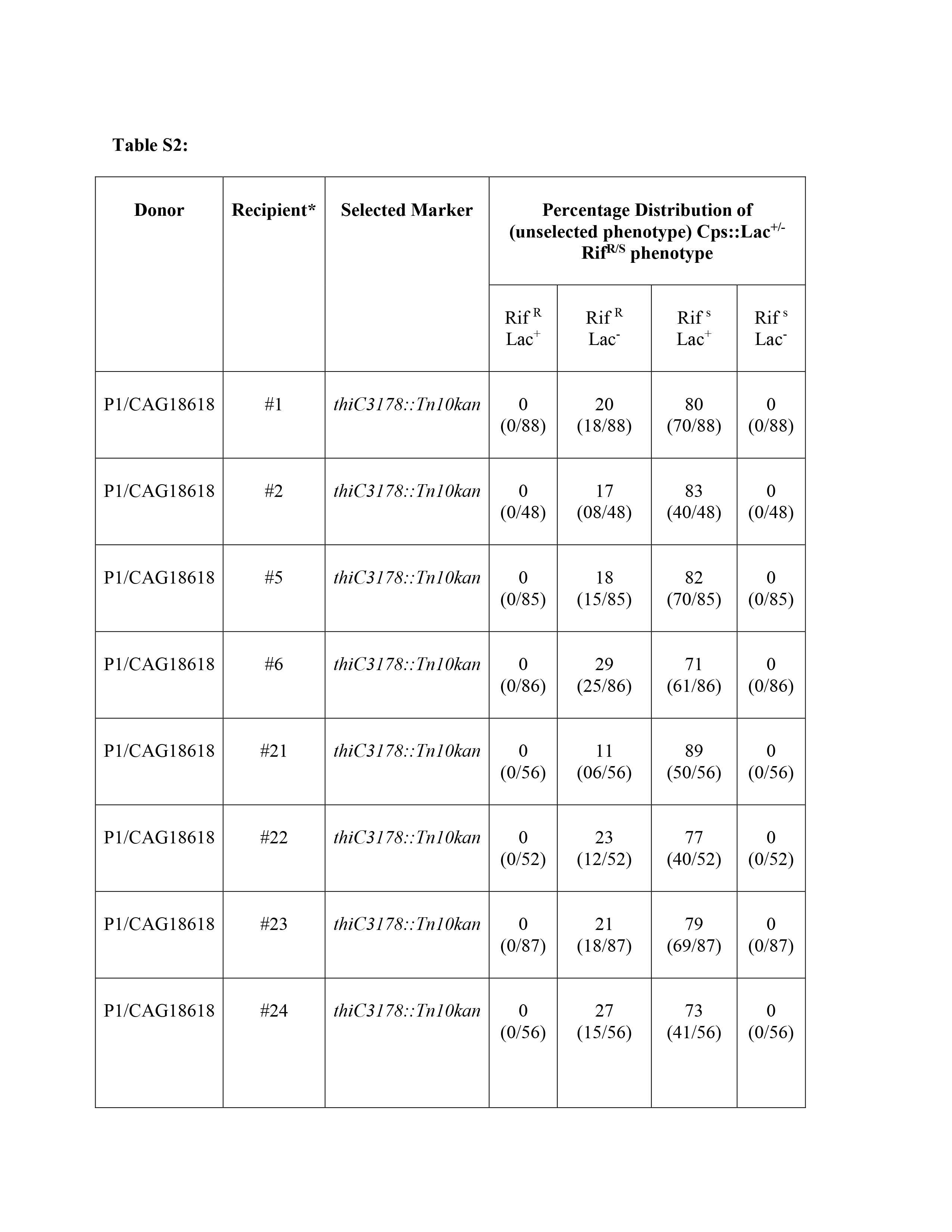

Supplement: Supplementary file 4 — Table S2. Percentage distribution of Cps::Lac+/− – RifR/S phenotype among KanR transductants obtained in the P1 transductional crosses involving P1 made of CAG18618 bearing KanR (thiC3178::Tn10kan) as donor and RifR mutants of SG20780 as recipients. [file mbo30004-0712-sd4.jpg]

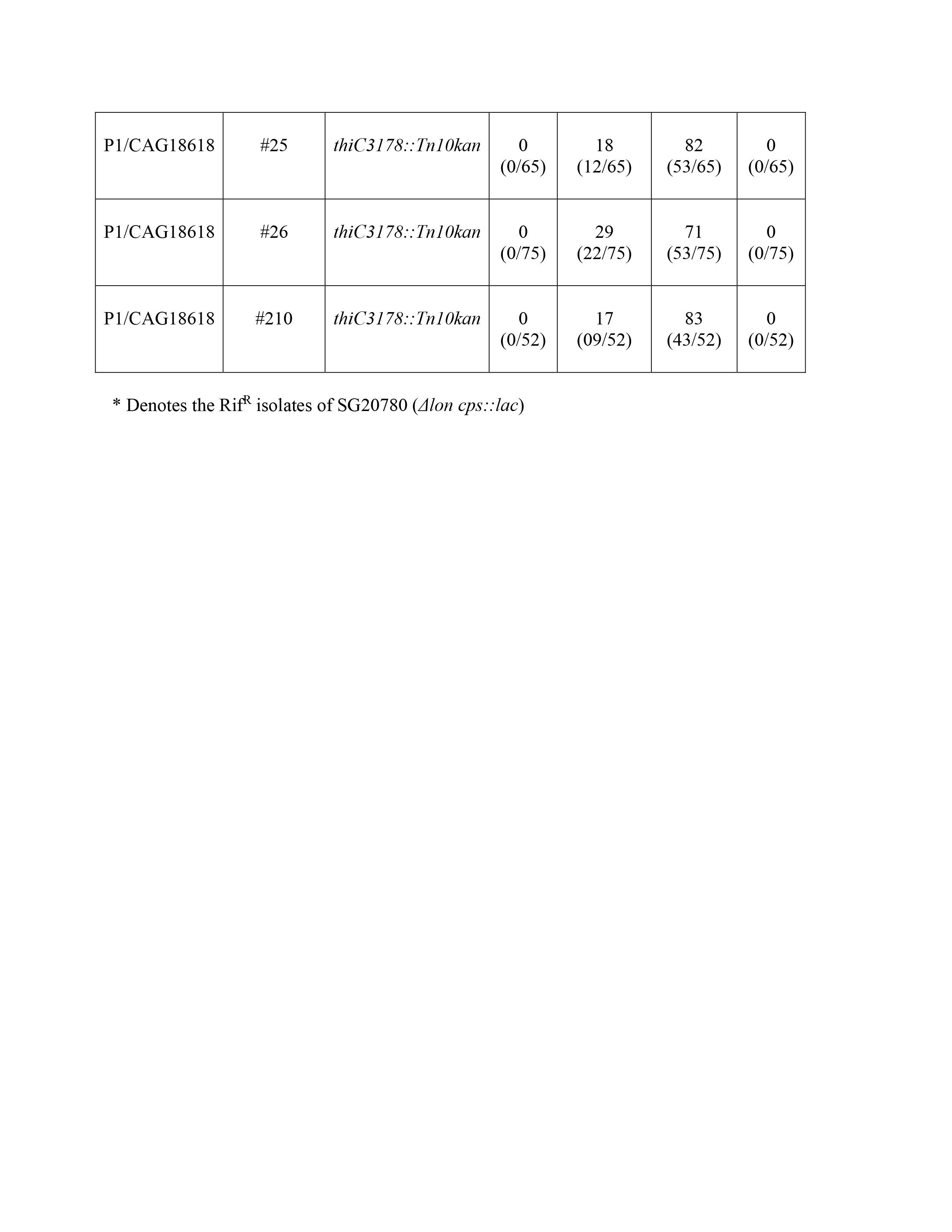

Supplement: Supplementary file 5 [file mbo30004-0712-sd5.jpg]
