# Supplementary material for: Suppression of capsule expression in Δlon strains of Escherichia coli by two novel rpoB mutations in concert with HNS: possible role for DNA bending at rcsA promoter
Source: Microbiologyopen. 2015 Sep 25;4(5):712–29. doi: 10.1002/mbo3.268 (PMC4618605; doi:10.1002/mbo3.268)
Supplement: Supplementary file 3 — Table S1. Distribution of Cps::Lac−/MMSR phenotype among spontaneous RifR mutants isolated from SG20780 (Δlon cps::lac). [file mbo30004-0712-sd3.pdf]

Table S1:

**Experiment No.**	**No. of Rif ^{R} colonies obtained in LB+Rif Agar Plates**	**No. of Lac^{-} colonies among Rif ^{R} mutants**	**No. of MMS^{R} colonies among Rif ^{R} mutants***
1.	40	1	1
2.	18	0	0
3.	84	1	0
4.	63	0	0
5.	70	2	0
6.	10	1	0
7.	17	2	0
8.	11	1	0
9.	21	0	0
10.	33	3	0
	Total	11/367	1/367

***** Since genetic analyses revealed that in the only one Rif^{R} MMS^{R} isolate obtained, the *rif* mutation does not play any role in eliciting the MMS^{R} phenotype, this aspect was not studied further (see text for more details).

Table S2:

Donor	Recipient*	Selected Marker	Percentage Distribution of (unselected phenotype) Cps::Lac$^{+/-}$ Rif$^{R/S}$ phenotype			
			Rif ^{R} Lac^{+}	Rif ^{R} Lac^{-}	Rif ^{s} Lac^{+}	Rif ^{s} Lac^{-}
P1/CAG18618	#1	*thiC3178::Tn10kan*	0 (0/88)	20 (18/88)	80 (70/88)	0 (0/88)
P1/CAG18618	#2	*thiC3178::Tn10kan*	0 (0/48)	17 (08/48)	83 (40/48)	0 (0/48)
P1/CAG18618	#5	*thiC3178::Tn10kan*	0 (0/85)	18 (15/85)	82 (70/85)	0 (0/85)
P1/CAG18618	#6	*thiC3178::Tn10kan*	0 (0/86)	29 (25/86)	71 (61/86)	0 (0/86)
P1/CAG18618	#21	*thiC3178::Tn10kan*	0 (0/56)	11 (06/56)	89 (50/56)	0 (0/56)
P1/CAG18618	#22	*thiC3178::Tn10kan*	0 (0/52)	23 (12/52)	77 (40/52)	0 (0/52)
P1/CAG18618	#23	*thiC3178::Tn10kan*	0 (0/87)	21 (18/87)	79 (69/87)	0 (0/87)
P1/CAG18618	#24	*thiC3178::Tn10kan*	0 (0/56)	27 (15/56)	73 (41/56)	0 (0/56)

P1/CAG18618	#25	*thiC3178::Tn10kan*	0 (0/65)	18 (12/65)	82 (53/65)	0 (0/65)
P1/CAG18618	#26	*thiC3178::Tn10kan*	0 (0/75)	29 (22/75)	71 (53/75)	0 (0/75)
P1/CAG18618	#210	*thiC3178::Tn10kan*	0 (0/52)	17 (09/52)	83 (43/52)	0 (0/52)

* Denotes the Rif^R isolates of SG20780 (*Δlon cps::lac*)

Figure S1

(A)

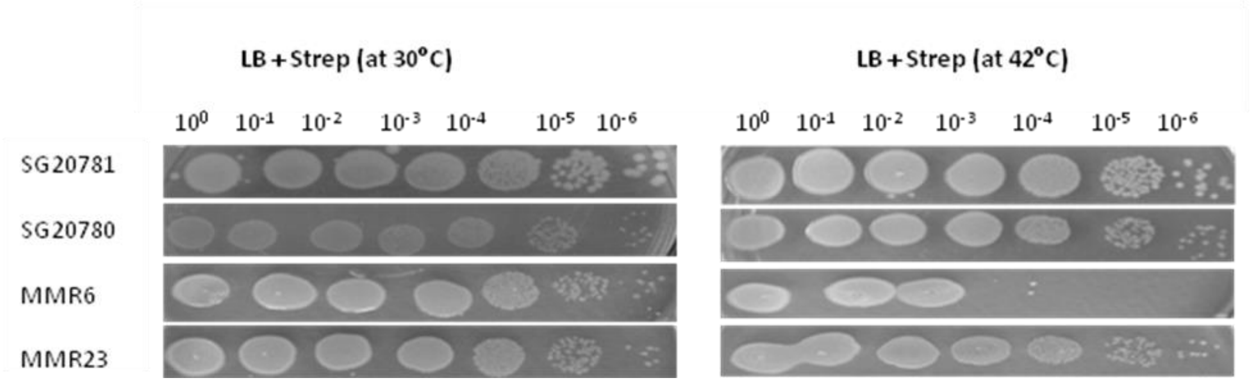
LB + Strep (at 30°C)
LB + Strep (at 42°C)
10^{0} 10^{-1} 10^{-2} 10^{-3} 10^{-4} 10^{-5} 10^{-6}
SG20781
SG20780
MMR6
MMR23

(B)

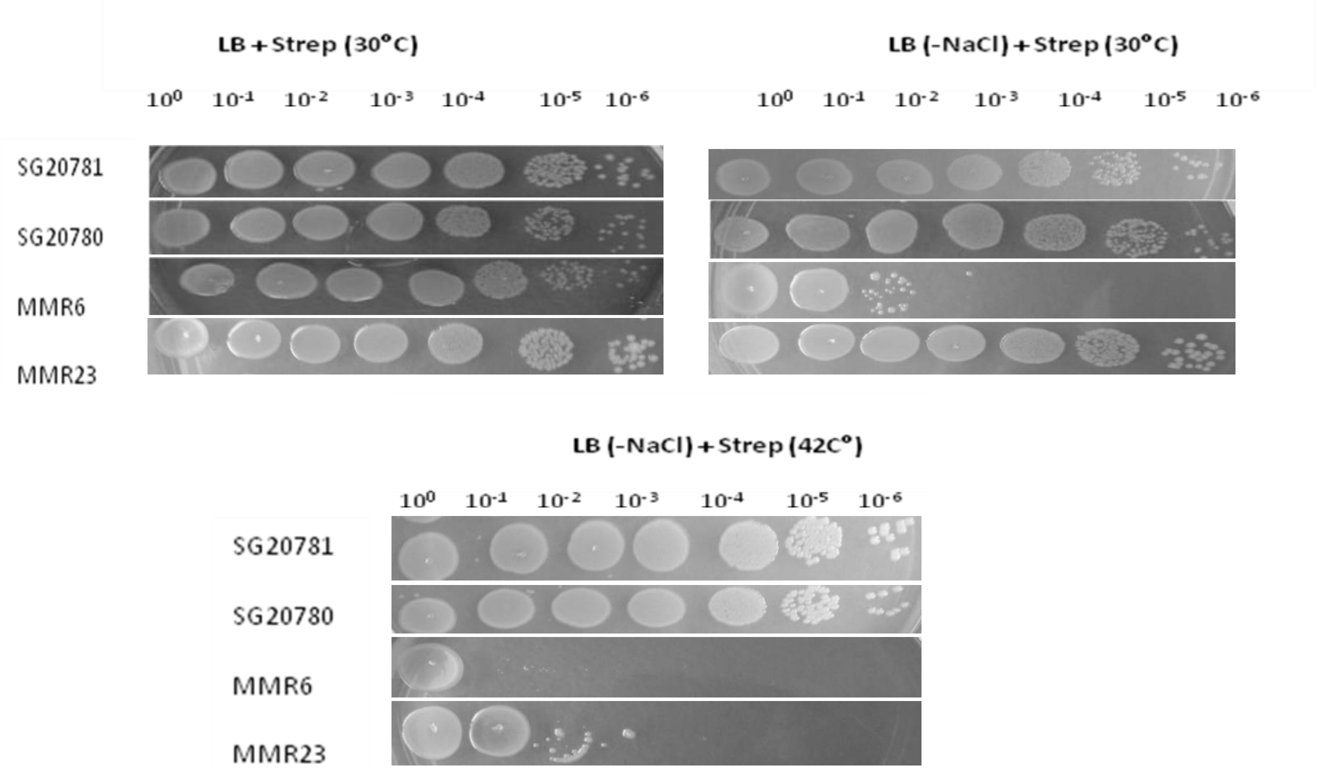
LB + Strep (30°C)
LB (-NaCl) + Strep (30°C)
LB (-NaCl) + Strep (42C°)
10^{0} 10^{-1} 10^{-2} 10^{-3} 10^{-4} 10^{-5} 10^{-6}
SG20781
SG20780
MMR6
MMR23

(C)

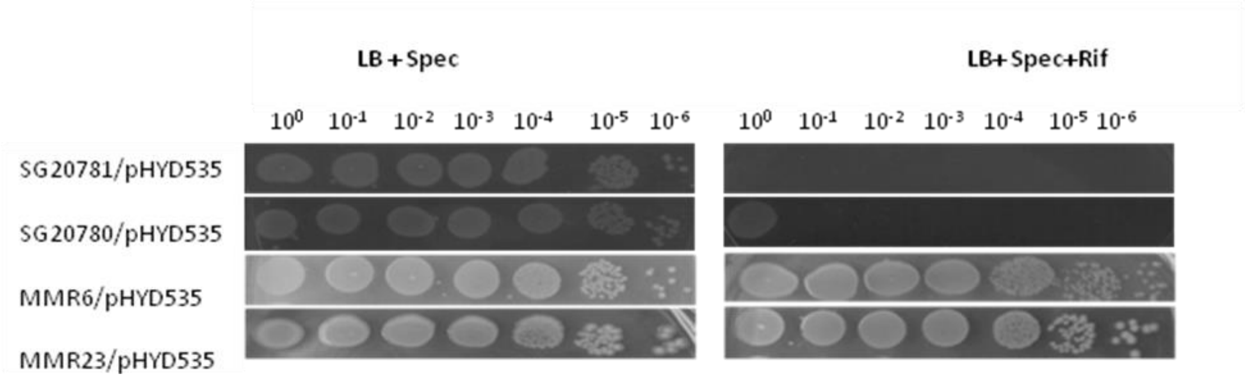
LB + Spec
LB+ Spec+Rif
10^0 10^-1 10^-2 10^-3 10^-4 10^-5 10^-6
10^0 10^-1 10^-2 10^-3 10^-4 10^-5 10^-6
SG20781/pHYD535
SG20780/pHYD535
MMR6/pHYD535
MMR23/pHYD535
